# In a Hospital Mortuary

**Published:** 1887-09-24

**Authors:** 


### IN A HOSPITAL MORTUARY.

By a Hospital Nurse.

I entered softly and stood pondering  
On life and death, until it seemed  
As though a vision passed before my sight,  
Of all the silent dead;  
Who, wearied with life's toiling and unrest,  
Lie here at last.  
The aged man, weary with weight of years,  
The child, whose little feet scarce crossed  
The threshold of our life,  
The young and strong in life's full energy,  
The good, the bad, the sinner and the saint,  
All sleep within this place.  
Tread gently, they who lie at rest  
Are in God's keeping, and His hand  
Has set His seal upon their quiet brows.  
Speak gently in the presence of His dead,  
Beggars, perchance, but yesterday, to-day in Paradise.  
Judge gently of the weak and erring souls,  
Thou knowest but a little, God knows all,  
All of His mercy thou canst never know.

